# American Dental Association

**Published:** 1883-12

**Authors:** 


### AMERICAN DENTAL ASSOCIATION—AGAIN.

"PLAIN WORDS ARE BEST."

There were some things which occurred at the last meeting of the American Dental Association which cannot be passed over in silence. They grow more aggravating as one reflects upon them. The quotation from an editorial in the INDEPENDENT PRACTITIONER, at the head of this article, encourages the writer in giving a few personal impressions which were made upon him, while a certain subject was under discussion.

I entered the parlors of the International Hotel, at Niagara Falls, where the Association held its meeting, just as that body had attacked the subject of Dental Education. In my time it has been my fate to hear a great deal of loose, rambling and pointless talk at such meetings, but never has it been my misfortune to listen to such nonsense and witless verbiage as was heard by that audience. While that subject was up for discussion, New York seemed to claim most, if not all, the attention of the speakers, and to growl at something that New York had or had not done was the rage of the hour. One New Jersey orator was especially eloquent in his denunciation. But what appeared to me the most unfortunate part of this whole display, was the utter lack of "Education," or even common information, on the part of those who were foremost in attacking the law of the State of New York. Their rantings only proved them deplorably ignorant of the law and its provisions. No reasonable explanation could be made of their tirade, unless it was their intention to talk against time, and in that they were eminently successful.

The dentists of New York are quite content with their law, and it is quietly, but surely accomplishing the objects that its originators intended, "improving and regulating the practice of dentistry in the State."

The law may not be perfect in every particular, but if the American Dental Association is dissatisfied with it, and wishes it changed, they are taking a very sure way *not* to accomplish their end.

And the Baby of the A. D. A. with the long name, "The National Association of Dental Examiners," takes up the cry, and makes a still worse mess of it, by recommending a law the whole burthen of which is to create a board of examiners *that will be entirely independent and beyond the control of any State society or other organization, and responsible to nothing but its own sweet will.* In so far as that "august" body copied the present law of the State of New York it did well, and if it had copied the provisions relating to the formation of State and district societies, and especially to the organization of the boards of dental examiners, it would have exhibited better judgment. Such board should always be elected by the State society, controlled by the State society, and responsible to that society for its every act, with no power, in and of itself to grant a diploma or certificate of qualification. The working of the New York law, which has been in successful operation for fifteen years, is simply this: "When a candidate presents himself before them with the proper credentials from the censors of the district society in which he resides," said credentials must state that the candidate "is of good moral character and professional attainments," "has been with some reputable dentist four years," "one year deducted for a complete course of lectures in a dental or medical college."

Then, and only then, has the New York State Board of Censors a right to examine him. "And if the inquiry and examination shall be satisfactory, *the board shall recommend him* to the State society for a diploma or certificate of qualification." If their report or recommendation is accepted by the State society, the "President in the presence of the State society," *not* the Board of Censors (their duty is, solely, to examine), issues the diploma or certificate of qualification.

That is—the Dental Society of the State of New York, reserves to itself the right to, at all times, control its Board of Censors, believing it will best serve the interests of all to have this power in the hands of the profession, guarded by the State society, rather than to delegate it to a few, as this law recommended by the "National Association of Dental Examiners" does.

New York can congratulate herself that she was not represented, and had no voice in promulgating that law.

Another striking point of contrast occurs between the recommendations of that conference of examiners, and the actual workings of the New York law. It has been notorious for years that the examinations of the New York board have been more rigid and more severe than any that have ever been made of the candidates for graduation in any dental college.

The proofs of this are seen, first, in the comparatively small number who have attained the degree—only about eighty in fifteen years, while some of the other State boards have turned out as many "certificates of qualification" in a single year; and secondly, hardly a year passes but some candidate who has received his degree from a dental college, but who very properly regards the State degree as of higher value, is so unfortunate as not to pass the examination and attain it.

The National Conference publishes its examination papers. No candidate can secure the degree of M. D. S. in the State of New York, until he has passed an ordeal three times more exacting than that entire list of questions indicates.

The law of New York now requires that *every* man beginning the practice of dentistry hereafter within its borders, shall possess a degree. It may be M. D., it may be D. D. S.; but if a man possessing neither comes from another State, even after long experience, he cannot practice until his degree is registered in the County Clerk's office of the county wherein he intends practicing. The wisdom of the New York law is shown in the provision it makes for men who, by long practice or otherwise, are thoroughly qualified dentists, but who have no degree.

How is it possible for any reasonable being to maintain for a moment that a degree hedged about with such difficulties is "adverse to the interests of the dental profession."

Vastly more pertinent would have been a resolution of the Association saying "that a certificate of *qualification* granted upon the examination papers of the Association of State Boards will be deemed "adverse to the interests of the dental profession."

"Whom the gods will destroy they first make mad."

The resolution of the American Dental Association above referred to, must have had some such fatality in its origin. But who would have believed that the Dean of the Pennsylvania Dental College had been so suddenly converted, and must make such marked exhibition of virtue.

How would the Dean and Faculty of the Pennsylvania Dental College have regarded a similar resolution emanating from a national society of dentists upon their action a few years since, when they invited a number of gentlemen to an informal conversation on dental topics at the close of a session, and forthwith their names appear *as graduates*, with the degree of D. D. S., not one of whom had attended a single course of lectures in that college or anywhere else.

The dentists of New York State are disposed to be liberal, and cultivate harmony, professional and social good feeling among their fellows, and are at all times ready to do their part in every honest endeavor to unite and secure a high and uniform standard of qualification. And I trust they will continue to do so, until this question of dental education has a firmer basis, a higher standard, and more attention is given to the qualifications necessary to obtain the degree of D. D. S.

What is most needed is some method by which the qualifications of each student shall be thoroughly *tested* before he can enter the ranks of the profession, an entire change in the manner of granting the degree, and one that will oblige students to seek the schools where they will be best taught, in lieu of those where they can get through the easiest. K. H.

---

### GOOD SOLDERS.

Hard Silver—Silver, 2 dwt.; copper, 9 grs.; zinc, 3 grs.

Soft Silver—Silver, 2 dwt.; copper, 8 grs.; zinc, 5 grs.

Hard Gold—Gold, 2 dwt.; copper, 9 grs.; silver, 3 grs.; zinc, 1 gr.

Soft Gold—Gold, 2 dwt.; copper, 8 grs.; silver, 5 grs.; zinc, 5 grs.

